# Vaccine cold chain management practice and associated factors among health professionals in Ethiopia: systematic review and meta-analysis

**DOI:** 10.1186/s40545-023-00560-1

**Published:** 2023-04-12

**Authors:** Abebaw Wasie Kasahun, Amare Zewdie, Solomon Shitu, Girma Alemayehu

**Affiliations:** 1 Department of Public Health, College of Medicine and Health Science, Wolkite University, Wolkite, Ethiopia; 2 Department of Midwifery, College of Medicine and Health Science, Wolkite University, Wolkite, Ethiopia

**Keywords:** Cold chain, Vaccine, Practice, Health professionals, Ethiopia

## Abstract

**Background:**

Administration of potent vaccine in a manner of well-maintained cold chain system is one of the public health focus areas in developing regions of the world. Health professionals’ adherence towards good vaccine cold chain management practices is an important element to ensure potent vaccine reached to users. Studies on health professionals’ practice on vaccine cold chain maintenance and associated factors in Ethiopia have shown wide variations. The aim of this systematic review and meta-analysis is to produce the overall/pooled prevalence of health professionals’ good vaccine cold chain management practice and to identify its associated factors in Ethiopia.

**Methods:**

Systematic review and meta-analysis was conducted on vaccine cold chain management practice and associated factors among health professionals in Ethiopia. Literature search was made on international data bases using medical subject heading and key words. Data were extracted using Microsoft excel and imported to STATA version 17 for analysis**.** Heterogeneity was checked using Cochrane Q test and I^2^ statistics. Weighted inverse variance random effect model was used to estimate the pooled level of good vaccine cold chain management practice among health professionals. Publication bias was checked using funnel plot and using Egger’s test.

**Results:**

A total of ten studies were included in the review. The overall/pooled prevalence of good vaccine cold chain management practice in Ethiopia is 27.48% with 95% CI (25.70–29.26). Having good knowledge on vaccine cold chain management AOR 2.27 95% CI (1.72–2.99), and have received on-job training AOR 6.64 95% CI (4.60–9.57) are factors positively associated with vaccine cold chain management practice among health professionals in Ethiopia.

**Conclusion:**

The overall/pooled prevalence of good vaccine cold chain management practice is much lower than the expected level. There is a need to plan on-job trainings for all vaccine handlers and other health professionals supposed to work on vaccination program.

**Supplementary Information:**

The online version contains supplementary material available at 10.1186/s40545-023-00560-1.

## Introduction

Though the world has gained notable achievement in child mortality reduction in the last two decades, there is still high under-five mortality. About ten million under-five children are dying due to preventable causes each year globally; of which the majority of deaths happened in developing regions [1, 2].

Concerted public health interventions have been implemented at different levels for maximizing child survival. Immunization is one of the most effective strategies for preventing infectious diseases and associated child deaths [3]. According to the World Bank report, the immunization program is saving over two million children’s lives annually in the world. Despite this fact, still vaccine-preventable diseases attribute 25% of under-five mortality worldwide [4, 5]. Despite the introduction of lifesaving vaccines, vaccine-preventable diseases (VPDs) still account for the death of more than half a million children under 5 years old every year in Africa representing 58% of global VPD-related deaths [6].

Vaccines are very sensitive biological molecules to a temperature that their potency could be irreversibly lost when exposed to out of the recommended range of temperature. The potency of the vaccines has to be maintained by the cold chain system that satisfies specific temperature requirements [7]. Therefore, vaccines must pass through a standard cold chain system from the manufacturing process to administration to ensure the effectiveness of the immunization program. Effective vaccination program requires the administration of potent vaccines in a manner of a well-maintained cold chain system and achieving high vaccination coverage. However, health workers seem to be overwhelmingly concerned with only raising vaccination coverage, whereas the focus on adhering to standard vaccine cold chain management did not get the attention it deserves in developing countries [8].

The World Health Organization recommends that vaccine should be stored in a refrigerator keeping the temperature reading between + 2 and 8 °C and transporting vaccines using vaccine carriers through properly prepared coolant packs at the health facility. Vaccine refrigerators, electronic refrigerator temperature loggers, and vaccine vial monitoring are essential logistics for maintaining the potency of vaccines [9].

Cold chain system consists of a series of storage and transport links, all designed to keep vaccines within an acceptable temperature range until it reaches the users. Improper storing and handling of vaccines can let vaccines to lose potency, fail to ignite immune responses, and be unable to prevent vaccine-preventable diseases [10].

The vaccine cold chain guideline recommends the following: the vaccine storage should be maintained in the temperature range of 2–8 °C, the use of minimum/maximum thermometers, temperature charts, and the shake test [11, 12]. Availability of cold chain equipment, knowledge of cold chain handlers, EPI (Expanded Program of Immunization) guideline utilization, education status, in-service training, and supervision are some of the factors affecting vaccine cold chain management practices [13, 14].

Adherence of health professionals to good vaccine cold chain management practices is an essential component effective vaccination program. However, health professionals’ practice on vaccine cold chain management systems is mixed across health sectors and the determinants are also different across different settings [15–17]. The level of good Vaccine cold chain maintenance system and temperature monitoring is still a major concern in developing countries where both coverage and quality of immunization service is suboptimal.

Ethiopia has gained a steady increment in coverage of all basic vaccination coverage. According to the national health and demographic health surveys, coverage of all basic vaccinations increased from 39% in 2016 to 43% in 2019 [18, 19]. Though significant progress has been made in increasing vaccine coverage in Ethiopia, VPD outbreaks are still being reported in different parts of the country [20, 21]. One of the speculations made for the occurrence of VPD is poor vaccine cold chain management practice at different levels of the health sector spanning from vaccine procurement, storage, handling, and transporting to users.

The findings of previous studies conducted on vaccine cold chain management practice and associated factors among health professionals in Ethiopia have shown wide variations in prevalence of good vaccine cold chin management practice. Factors associated with good vaccine cold chain management practice are also inconsistent across different studies [15–17, 22, 23] which is difficult to utilize for crafting evidence-based public health interventions. Therefore, this systematic review and meta-analysis is aimed to produce the overall/pooled prevalence of good vaccine cold chain management practice and to identify significant predictors of good vaccine cold chain management practice among health professionals in Ethiopia.

## Methods

### Study design and setting

The PROSPERO website (http://www.library.ucsf.edu/) has been checked whether the title is previously addressed or whether an ongoing review exists to avoid duplications. Accordingly, we found no registered published and/or ongoing systematic review and meta-analysis on vaccine cold chain management practice and associated factors among health professionals in Ethiopia. The protocol of this systematic review and meta-analysis is registered on PROSPERO with registration number CRD42023400254. A systematic review and meta-analysis were conducted to determine the overall/pooled prevalence of good vaccine cold chain management practice and to identify its determinants among health professionals in Ethiopia. The review was conducted following the preferred reporting items for systematic review and meta-analysis.

### Search strategies and source information

Firstly, preliminary search was done using medical subject headings. Secondly, keywords were developed using keywords from retrieved articles on the preliminary search. Finally, medical subject headings and keywords were used to search articles on medical and health sciences research databases and other search engines. Furthermore, librarians were consulted to find unpublished research works on our area of interest for this review. Databases including PubMed, Scopus, African Journals Online, Web of Science, Google Scholar, and Google were used to find research articles on vaccine cold chain management practice and associated factors among health professionals in Ethiopia. Furthermore, a hand search was made on references of retrieved articles to find all eligible articles for this review.

Search terms were designed using PICO guidelines. Searching for articles was done using medical subject headings (MeSH) and key terms through online databases. Boolean operators including “AND” and “OR” were used to link MeSH and the keywords for searching purposes. Accordingly, the developed search terms consist of “Vaccine Cold chain” OR “cold chain” AND “practice” OR “maintenance” OR “handling” AND “determinants” OR “associated factors” OR “predictors” AND “Ethiopia”.

### Eligibility criteria

Studies reporting the prevalence of good vaccine cold chain management practice and associated factors among health professionals in Ethiopia were included in this review. Published research articles and unpublished researches including preprints and grey literature written in the English language were eligible regardless of the study design. Articles published at any time until the end of our search (February 2, 2023) were included in this review. Articles that did not report the outcome variables or articles with unrelated outcome variable to the interest of this review were excluded. Furthermore, articles without full abstracts, commentaries, editorials, letters, and anonymous reports were excluded.

### Outcome measurement

The prevalence of good vaccine cold chain management practice and factors associated with good vaccine cold chain management practice are the two outcome variables of this review. Vaccine cold chain is said to be properly maintained if and only if there is proper temperature monitoring (twice daily monitoring), proper review of vaccine vial monitoring to check the exposure of vaccine to heat, undertaking shake test to check the exposure of vaccine to freezing temperature, adherence to the first expiry first out vaccine flow in distributing vaccine to users, regular cleaning and defrosting of refrigerator/fridge ice and timely cold chain equipment maintenance during breakage as per WHO recommendation. Good vaccine cold chain management practice was assessed by soliciting health professionals on the aforementioned vaccine handling items. The total vaccine cold chain management practice score was obtained after adding each response (if they provide the correct practice response for the item, they got 1 or else 0 points). The mean score of respondents on vaccine cold chain management practice assessment items was computed, and health professionals who scored below the mean score were labeled as having poor practice, while those who scored the mean and above the mean value were labeled as having good practice on vaccine cold chain management.

### Data extraction

Data were extracted using the Joanna Briggs Institute data extraction form for observational studies [24]. Firstly, identified articles were imported to EndNote X6 to identify and remove duplicated articles. Secondly, important data were extracted using the prepared data extraction format independently by two authors (AZ and AW). For the first outcome of the review, the extracted data include the primary author name, study year, publication status, publication month, publication year, study design, sample size, prevalence, study region, determinant factors, and quality of the study. Data were extracted using 2 by 2 tables for the second objective of this review (associated factors of health professionals’ practice on vaccine cold chain management). All data extraction activity was done by two authors (AW and AZ). Finally, data analysis was done by STATA software version 17.

### Quality assessment

Modified Newcastle Ottawa Quality Assessment Scale for cross-sectional studies was used to assess the quality of studies [25]. Two authors (AW and AZ) assessed the quality of each study using the aforementioned quality assessment scale. The quality assessment scale includes methodological quality, sample selection, sample size, outcome measurement, and statistical analysis used. In cases of disagreement between the two authors the third author (GA) is involved in the resolution (Additional file 1).

### Data processing and analysis

Data were extracted using the Microsoft Excel spreadsheet format and imported to STATA software version 17 for analysis**.** Heterogeneity among studies was checked using the Cochrane *Q* test and *I*^2^ statistics. The level of heterogeneity among studies is quantified by *I*^2^ statistics. Accordingly, if the result of *I*^2^ is 0–40% it is mild heterogeneity, 30–60% would be moderate heterogeneity, 50–90% would be substantial heterogeneity; and 75–100% would be considerable heterogeneity [26]. The weighted inverse variance random effect model was used to estimate the pooled prevalence of good vaccine cold chain management practice in Ethiopia. The random effect model was used due to the observed considerable heterogeneity (*I*^2^ = 98.4%) among studies. A fixed effect model would have been used if studies were homogeneous. Forest plot was used to illustrate the pooled prevalence of good vaccine cold chain management practice with 95% CI. Publication bias was checked visually using a funnel plot and statistically using Egger’s regression test, with *p* < 0.05 indicating significant publication bias. Sensitivity analysis was done to estimate the effect of a single study on the overall estimate of the prevalence of good vaccine cold chain management practice and sub-group analysis was done using regions where the studies are conducted.

## Results

**Fig. 1:**
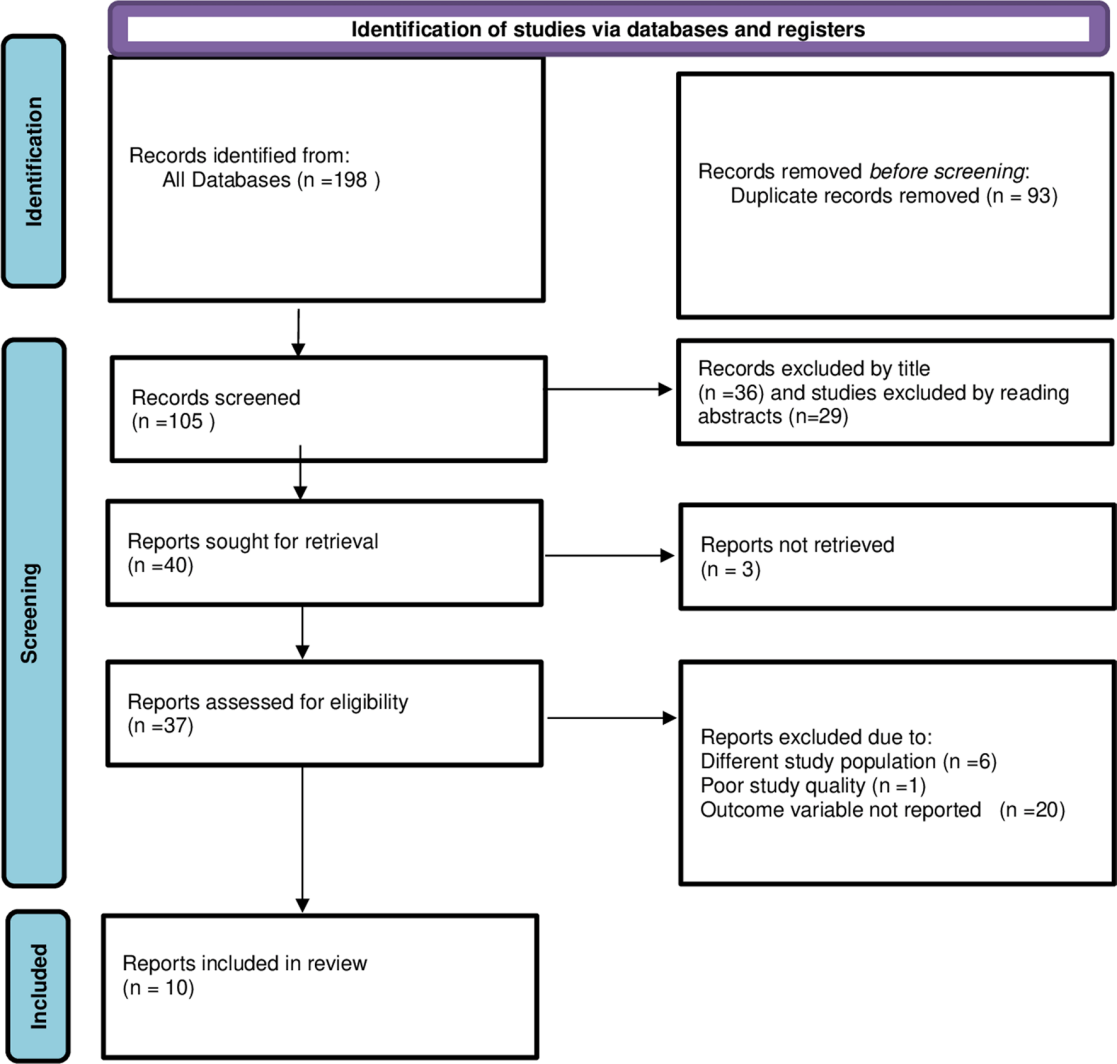
Flowchart illustrating selection of studies for systematic review and meta-analysis on vaccine cold chain management practice and associated factors among health professionals in Ethiopia, 2023

The search strategy yielded a total of 198 articles about vaccine cold chain management practices in Ethiopia. Duplicates (93 articles) identified by endnote software and removed. From the remaining 105 articles 65 articles were excluded by reading the titles. Of the remaining 40 articles sought for full article retrieval, 37 full articles were retrieved and 3 articles were excluded because of unable to retrieve the full article. Thirty-seven articles were assessed for eligibility of which 27 articles were excluded due to reasons explained in Fig. 1. Accordingly, ten articles fulfilled the inclusion criteria and were included in the final systematic review and meta-analysis. Of the included ten studies three were from the Amhara region of Ethiopia, two were from SNNPR (Southern Nations and Nationalities Region) of Ethiopia; two were from the Oromia region of Ethiopia, and Tigray region, Addis Ababa, and nationwide contribute one article each. All of the included studies have used cross-sectional study design and included sample size ranges from 41 to 632 health professionals (Table 1).

**Table 1:** Characteristics of included studies for systematic review and meta-analysis on vaccine cold chain management practice and associated factors among health professionals in Ethiopia, 2023

Author	Study year	Study region	Study design	Sample size	Prevalence of good practice (%)	Study quality
Asres et al. [16]	2019	Nationwide	Cross-sectional	632	36.5	Good
Feyisa [15]	2021	Oromia	Cross-sectional	41	24.4	Good
Feyisa et al. [27]	2022	SNNPR	Cross-sectional	140	41	Good
Mohammed et al. [28]	2021	Amhara	Cross-sectional	127	48.8	Good
Berhanu et al. [29]	2009	Addis Ababa	Cross-sectional	83	81.9	Good
Woldemichael et al. [30]	2018	Oromia	Cross-sectional	183	7.65	Good
Bogale et al. [22]	2019	Amhara	Cross-sectional	60	58.33	Good
Erassa et al. [31]	2023	SNNPR	Cross-sectional	136	61.03	Good
Esubalew et al. (unpublished) [32]	2020	Amhara	Cross-sectional	396	11.36	Good
Gebretnsae et al. [23]	2022	Tigray	Cross-sectional	50	46.00	Good

### Vaccine cold chain management practice among health professionals in Ethiopia

**Fig. 2:**
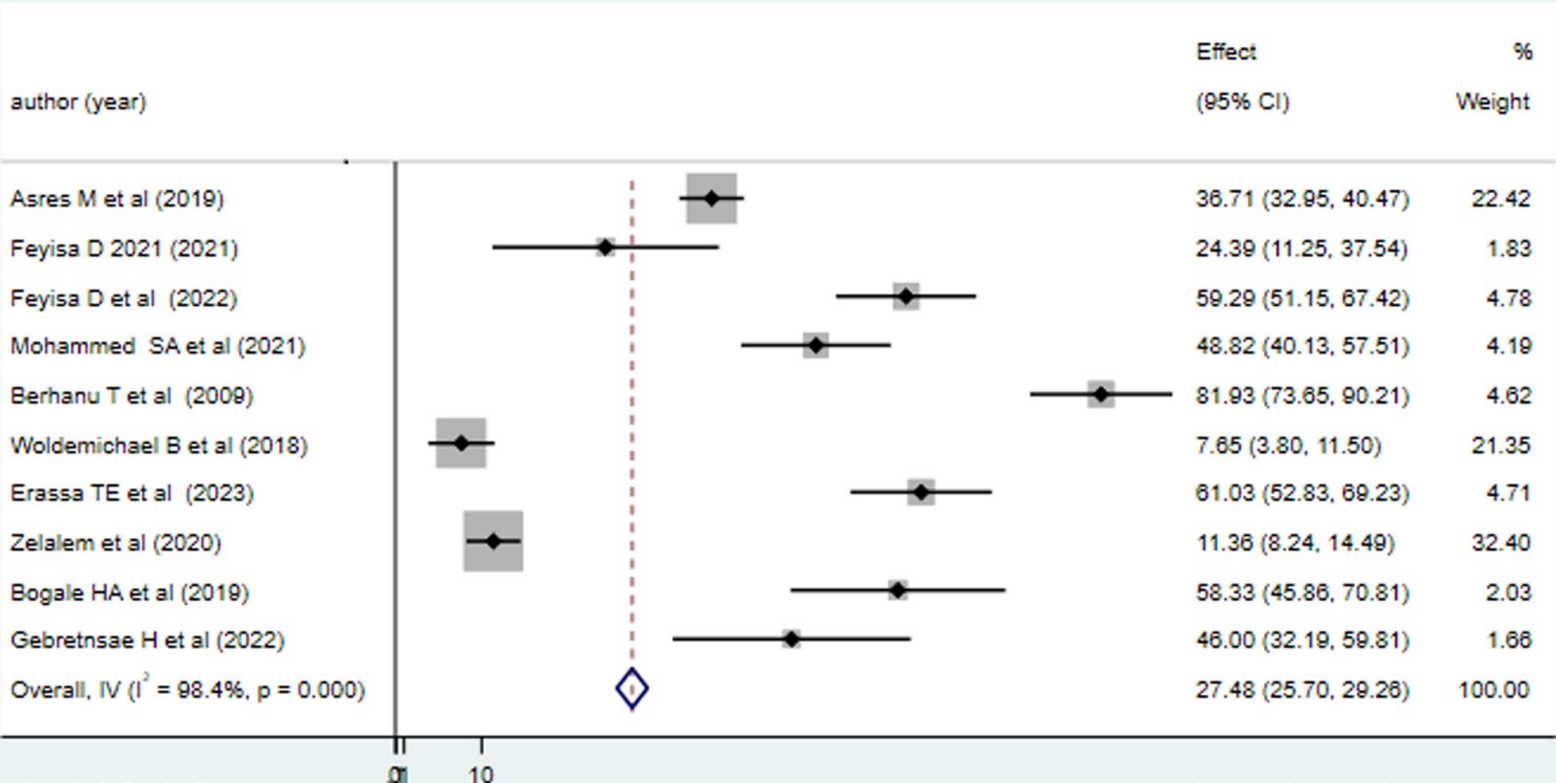
Pooled level of good vaccine cold chain management practice among health professionals in Ethiopia, 2023

The overall/pooled prevalence of good vaccine cold chain management practice among health professionals in Ethiopia is 27.48% with a 95% CI (25.70–29.26). A considerable level of heterogeneity is observed with *I*^2^ statistics (*I*^2^ = 98.4%, *p* = 0.000). The finding is summarized using a forest plot presented below in Fig. 2. Based on the findings of substantial heterogeneity sub-group analysis was done using regions where the studies are conducted.

### Publication bias

**Fig. 3:**
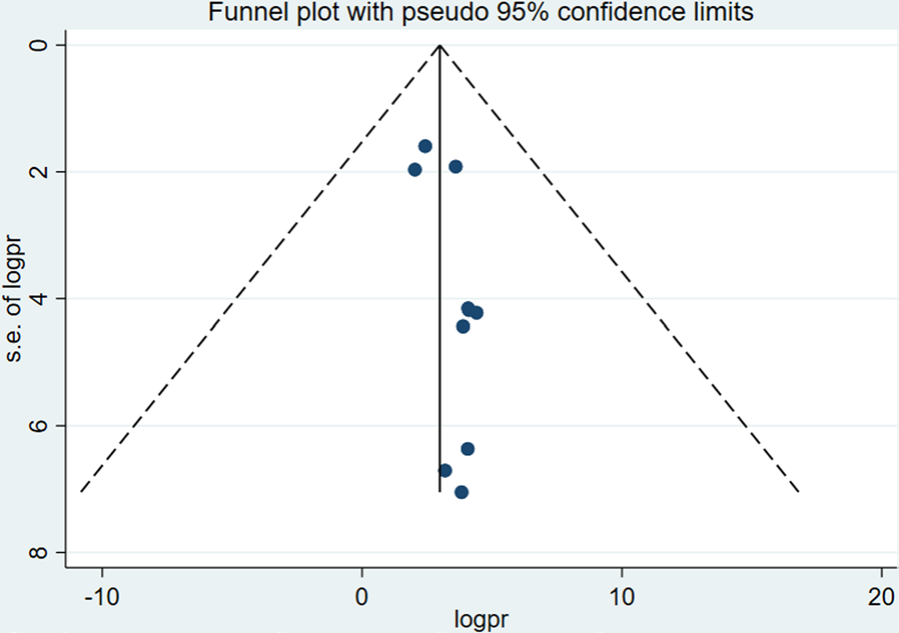
Funnel plot showing the symmetric distribution of articles on vaccine cold chain management practice and associated factors among health professionals in Ethiopia, 2023

Funnel plot and Egger’s regression test were used to assess whether publication bias is observed or not, accordingly, Egger’s test was not statistically significant (*p* = 0.48) which rule out the presence of publication bias. Furthermore, the funnel plot is symmetrical which confirms the absence of publication bias (Fig. 3).

### Sub-group analysis of health professionals’ practice on vaccine cold chain management

**Fig. 4:**
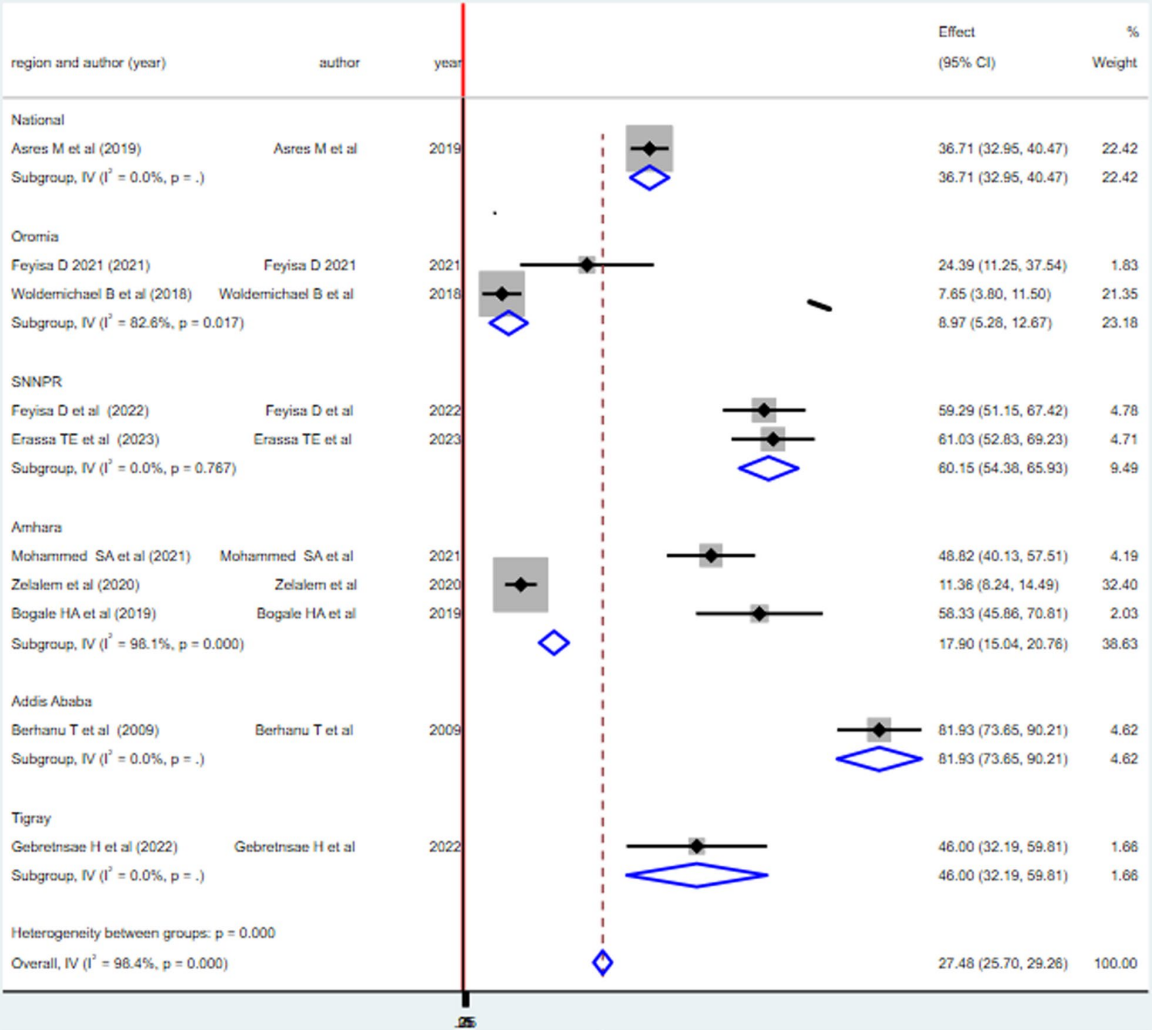
Forest plot illustrating sub-group analysis of vaccine cold chain management practice among health professionals by region in Ethiopia, 2023

The sub-group analysis showed that the overall prevalence of good vaccine cold chain management practice is highest in Addis Ababa with 81.93% 95% CI (73.65–90.21) and lowest in the Oromia region with an overall/pooled prevalence of good vaccine cold chain management practice of 8.87% 95% CI (5.26–12.67) (Fig. 4).

### Sensitivity analysis

**Fig. 5:**
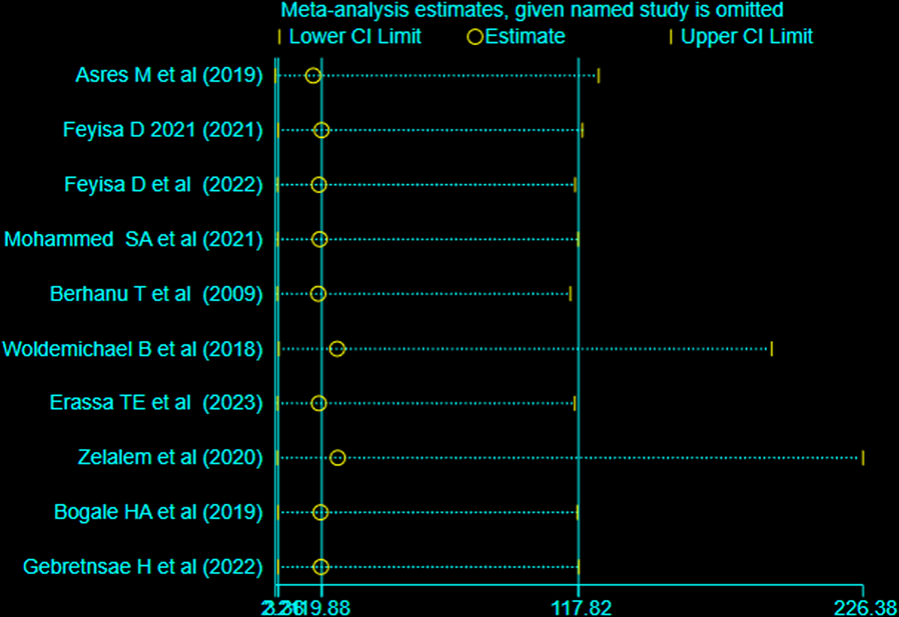
Sensitivity analysis of studies included for systematic review and Meta-analysis on vaccine cold chain management practice and associated factors among health professionals in Ethiopia, 2023

A random effect model finding illustrated that no single study has influenced the overall/pooled prevalence of health professionals’ vaccine cold chain management practice in Ethiopia (Fig. 5).

### Factors associated with vaccine cold chain management practice among health professionals

Health professionals who received on-job training on vaccine cold chain management and those who have good knowledge on vaccine cold chain management are factors positively associated with health professionals’ practice on vaccine cold chain management in Ethiopia.

Health professionals who have received on-job training are almost two times more likely to have good vaccine cold chain management practice compared to those who did not get on-job training with a 95% CI 1.94 (1.39–2.48). Health professionals who have good knowledge of vaccine cold chain management are 18% more likely to engage in good vaccine cold chain management practice compared to those who do have not satisfactory knowledge with a 95% CI 1.18 (1.16–2.47) (Table 2).

**Table 2:** Factors associated with vaccine cold chain management practice among health professionals in Ethiopia, 2023

Variable	Authors	AOR	95%CI	Pooled OR	95% CI of pooled OR
Health professionals who have got on-job training on cold chain	Erassa et al.	1.86	1.36–9.84	1.94	1.39–2.48
	Esubalew et al.	12.28	6.33–23.32		
	Gebretnsae et al.	4.20	1.19–14.83		
Good knowledge on cold chain management	Erassa et al.	3.02	1.2–7.4	1.18	1.16–2.47
	Gebretnsae et al.	10.97	2.67–45.07		
	Bogale	15.4	4.3–55.9		

## Discussion

This systematic review and meta-analysis is aimed to determine the pooled prevalence of good vaccine cold chain management practice and its associated factors among health professionals in Ethiopia. Accordingly, a little more than one-quarter of health professionals [27.48% with 95% CI (25.70–29.26)] have good vaccine cold chain management practices in Ethiopia. The sub-group analysis showed that the prevalence of good vaccine cold management practice is highest in Addis Ababa and lowest in the Oromia region of Ethiopia. The variation is due to the fact that Addis Ababa is the capital of the country and it is most likely to have well-established cold chain infrastructure including well-trained health care practitioners; whereas Oromia region is predominantly rural settings including the pastoral areas in which its health care infrastructures are not well designed to exercise good vaccine cold management.

The pooled good vaccine cold chain management practice is consistent with the level of good vaccine cold chain management practice in Mozambique where three-quarters of health professionals have inappropriate practices to maintain vaccine potency [33]. However, it is higher than the level of health professionals’ good vaccine cold chain management practice in Malaysia where only 5.6% of health professionals were adherent to the appropriate vaccine cold chain management practice [34]. The difference could be due to the variation in data collection technique in which the study in Malaysia used inspection/observation to collect data, whereas the majority of studies included in this systematic review and meta-analysis have used only self-reported responses of health professionals on vaccine cold chain management. The later data collection approach is more likely to overestimate the level of good vaccine cold chain management practice due to respondents’ bias. Corroborating what has been reported through observation provides relatively lower estimate of good vaccine cold chain management practice. Furthermore, it has to be kept in mind that the findings from Malaysia is taken from a single study, unlike the finding of this review which is the pooled prevalence combined from many studies in Ethiopia.

On the other end of the spectrum, studies in Oyo state of Nigeria, India and Ghana have reported a higher levels of good vaccine cold chain management practices than the finding of this study [35–37]. The observed difference could be due to the difference in the study population. For instance, the majority of the study population in Nigeria has received on-the-job training on the vaccine cold chain. The level of national interventions on vaccine cold chain management and the variation in vaccine cold chain infrastructures among study settings might be the reason for the observed difference in vaccine cold chain management practice.

Health providers with good knowledge of vaccine cold chain management and health professionals who have received training were significant predictors of good vaccine cold chain management practice and associated factors among health professionals in Ethiopia.

Health professionals who have good knowledge of vaccine cold chain management are more likely to have good vaccine cold chain management practices compared to those who have no satisfactory knowledge. This is obvious due to the fact that good knowledge helps health professionals to be adherent to the standard vaccine handling and management practices. This finding is consistent with studies conducted in Nigeria and Mozambique where knowledge is found to be a significant predictor of good vaccine cold chain management practices [33, 36].

Health professionals who have got on-job training on expanded vaccine cold chain management are about two times more likely to have good vaccine cold chain management practices compared to health professionals who didn’t get on-job training. It is known that on-job training is helpful to acquire the current state of the art in health care including vaccine handling; hence health professionals who have received on-job training are more likely to recognize evidence-based vaccine handling practices which will help them to refresh and sustain the standard vaccine cold chain management practices. This finding is consistent with studies conducted in Nigeria, Turkey, Yemen and, Malaysia which prove the effectiveness of on-job training of health professionals in improving their knowledge and practices of vaccine cold chain management [36, 38–40]. Despite this review has generated important findings for public health practitioners; it should be understood that it is not without limitations. The major drawback of this review is that included studies did not use identical assessment items for the outcome variable; hence the observed inconsistency of findings among included studies might be artificial.

## Conclusions

The pooled prevalence of good vaccine cold chain management practice among health professionals is suboptimal in Ethiopia. It can be speculated that the potency of vaccines reached to users is highly compromised due to poor vaccine handling and management practices of health professionals. Having good knowledge of vaccine cold chain management and having on -job training on vaccine cold chain management were significant predictors of good vaccine cold chain management practice. The finding calls for an urgent intervention to improve health professionals’ knowledge and practice on vaccine cold chain management through continuous on-job training. Furthermore, it is also important to avail cold chain management guidelines at each health facility so that health professionals would use them to refine their knowledge and practices towards standard vaccine cold chain management.

## Supplementary Information

**Additional file 1: **Newcastle Ottawa quality assessment of prevalence studies.

## Data Availability

The result of this systematic review and meta-analysis was extracted from the data gathered and analyzed based on the stated methods and materials. All the relevant data are within the paper.

## Abbreviations

EPI: Expanded Program on Immunization
PICO: Population, Intervention, Comparison, Outcome
PRISMA: Preferred Reporting Items for Systematic Reviews and Meta-Analyses
MeSH: Medical subject heading
SNNPR: South Nations, Nationalities and People Region
VPDs: Vaccine-preventable diseases
WHO: World Health Organization
