# Supplementary material for: Vaccine cold chain management practice and associated factors among health professionals in Ethiopia: systematic review and meta-analysis
Source: J Pharm Policy Pract. 2023 Apr 12;16:55. doi: 10.1186/s40545-023-00560-1 (PMC10099644; doi:10.1186/s40545-023-00560-1)
Supplement: Supplementary file 1 — Additional file 1: Newcastle Ottawa quality assessment of prevalence studies. [file 40545_2023_560_MOESM1_ESM.docx]

**Newcastle Ottawa quality assessment of prevalence studies**

| **Included Studies** | **Assessment criteria** | | | | | | | |  |
| --- | --- | --- | --- | --- | --- | --- | --- | --- | --- |
|  | Representativeness of the sample | Sample size: | Non-respondents | Ascertainment of the exposure (risk factor): | The subjects in different outcome groups are comparable, based on the study design or analysis. Confounding factors are controlled | Assessment of the outcome: | Statistical test | total | Remark |
| Asres M et al | 1 | 1 | 1 | 1 | 1 | 2 | 1 | 8 | good |
| Berhanu T et al | 1 | 1 | 1 | 2 | 1 | 2 | 1 | 9 | good |
| Feyisa D et al | 1 | 1 | 1 | 1 | 1 | 2 | 1 | 8 | good |
| Mohammed SA et al | 1 | 1 | 1 | 2 | 1 | 2 | 1 | 9 | good |
| Bogale HA et al | 1 | 1 | 1 | 1 | 1 | 2 | 1 | 8 | good |
| Woldemichael B et al | 1 | 1 | 1 | 1 | 1 | 2 | 1 | 8 | good |
| Feyisa D | 1 | 1 | 1 | 2 | 1 | 2 | 1 | 9 | good |
| Erassa TE et al | 1 | 1 | 1 | 2 | 1 | 2 | 1 | 9 | good |
| Esubalew Z et al(unpublished) | 1 | 1 | 1 | 2 | 1 | 2 | 1 | 9 | good |
| Gebretnsae H et al | 1 | 1 | 1 | 2 | 1 | 2 | 1 | 9 | good |
